# Socio‐Occupational Functioning after Subthalamic Deep Brain Stimulation in Parkinson's Disease

**DOI:** 10.1002/mdc3.70661

**Published:** 2026-05-01

**Authors:** Gabriele Imbalzano, Elisa Montanaro, Martina Giacchero, Claudia Ledda, Alberto Romagnolo, Mario Giorgio Rizzone, Leonardo Lopiano, Maurizio Zibetti

**Affiliations:** ^1^ Department of Neuroscience “Rita Levi Montalcini” University of Turin Turin Italy; ^2^ SC Neurologia 2U, AOU Città della Salute e della Scienza Turin Italy; ^3^ Clinical Psychology Unit, AOU Città Della Salute e Della Scienza di Torino Turin Italy; ^4^ Department of Psychology University of Turin Turin Italy

**Keywords:** deep brain stimulation, GBA variants, occupational outcomes, Parkinson's disease, sex, social functioning, subthalamic nucleus

## Abstract

**Background:**

Socio‐occupational functioning in patients with Parkinson's disease (PD) treated with subthalamic nucleus deep brain stimulation (STN‐DBS) is not fully captured by standard motor and quality‐of‐life scales.

**Objectives:**

To characterize patient‐reported socio‐occupational functioning after STN‐DBS and explore associated clinical and demographic factors.

**Methods:**

Thirty‐four PD patients were assessed 9–18 months postoperatively using a semi‐structured interview covering socio‐occupational domains. A composite mean score (Q_mean) was computed as a descriptive index, with clinical associations explored using univariate screening and multivariate linear regression.

**Results:**

The Q_mean score following STN‐DBS was 7.66 ± 0.86 (range 6.00–9.57). In exploratory multivariate analysis, female sex (n = 7) was associated with higher functioning (*P* = 0.003), whereas GBA variants (n = 5) with lower functioning (*P* = 0.007).

**Discussion:**

Perceived socio‐occupational functioning was overall satisfactory at postoperative assessment, with limited association with conventional motor/neuropsychiatric measures, and greater association with individual factors such as sex and genetic background. These exploratory findings support inclusion of socio‐occupational perspectives in DBS outcome evaluation.

Deep brain stimulation of the subthalamic nucleus (STN‐DBS) is a well‐established intervention for Parkinson's disease (PD) with motor complications, improving motor fluctuations, dyskinesias, activities of daily living (ADL), and quality of life (QoL).^1^ However, socio‐occupational functioning after STN‐DBS remains less well characterized. Most studies have focused on motor and global QoL outcomes, whereas return to work, role participation, and social reintegration have received comparatively little attention so far.

The ability to maintain or resume meaningful social and occupational roles is a major determinant of well‐being and QoL after DBS, yet it is not adequately captured by standard motor, neuropsychiatric, or QoL scales, and no validated instruments exist for assessing these domains in PD. This has contributed to limited and fragmented evidence regarding real‐life functional outcomes after STN‐DBS. Previous studies suggest that, despite clinical improvement, many patients do not return to previous employment or social activities after surgery, while social relationships and marital satisfaction generally remain stable.^2^, ^3^, ^4^

The EARLYSTIM trial showed that STN‐DBS may enhance social, occupational, and psychosocial functioning, particularly in younger patients with early motor complications.^5^ However, such improvements do not necessarily translate into higher employment rates or full professional reintegration, suggesting that non‐motor and contextual factors (eg, mood, cognition, or external occupational constraints) may limit real‐life outcomes.

The present study aimed to evaluate socio‐occupational functioning 9–18 months after STN‐DBS and to explore associated demographic and clinical factors.

## Methods

We included consecutive patients with idiopathic PD who underwent bilateral STN‐DBS at Turin University Hospital between April 2021 and November 2023. Surgical procedure and targeting protocol have been described previously.^6^ Patients were eligible if complete pre‐ and post‐operative data (9–18 months after surgery) were available and if they agreed to participate in a semi‐structured socio‐occupational interview. We excluded cases with incomplete follow‐up or clinically evident postoperative dementia. The study complied with the Declaration of Helsinki and was approved by the local ethics committee. All patients provided written informed consent.

### Clinical and Neuropsychological Assessment

Demographic data, disease duration, and levodopa equivalent daily dose (LEDD) were collected as routine pre‐ and post‐operative evaluations.^7^ Motor severity and complications were assessed with the Movement Disorder Society‐Unified Parkinson's Disease Rating Scale (MDS‐UPDRS) parts I‐IV. MDS‐UPDRS III was assessed in OFF‐medication state (OFF‐MED, after overnight withdrawal of antiparkinsonian therapy) and ON‐medication state (ON‐MED), with ON‐stimulation during post‐operative evaluation, including an axial sub‐score (sum of MDS‐UPDRS items 3.1, 3.3, 3.9, and 3.10–3.13).^8^ Cognitive status was screened with the Mini Mental State Examination; mood, anxiety, apathy and QoL were measured using the Beck Depression Inventory (BDI), State–Trait Anxiety Inventory (STAI‐X1, STAI‐X2), Marin Apathy Scale (AS) and PD Questionnaire (PDQ‐39). As part of the pre/post‐surgical evaluation, all patients underwent genetic testing for major PD‐associated variants (*GBA, LRRK2, DJ1, PINK1, SNCA*, and *PRKN*).

### Socio‐Occupational Interview

In the absence of validated PD‐specific instruments for socio‐occupational functioning, we developed an ad hoc semi‐structured interview administered alongside the clinical and neuropsychological assessment (Supplementary Materials File S1). It covered occupational status and satisfaction, domestic activities, social and family relationships, leisure and hobbies, daily routine and autonomy, and general satisfaction with DBS outcomes, using both dichotomous (yes/no) and numerical rating (0–10) items (0 indicated “very poor/not satisfied,” while 10 “excellent/fully satisfied”). The interview was administered once during the postoperative follow‐up by a neuropsychologist trained in movement disorders and DBS, who also conducted routine pre‐ and postoperative neuropsychological assessments, providing a cross‐sectional assessment of perceived socio‐occupational functioning.

### Statistical Analysis

Continuous variables are reported as mean ± standard deviation and categorical variables as frequencies. Pre‐ and postoperative comparisons were performed using Wilcoxon signed‐rank tests. No missing data were present in the analyzed variables, as inclusion required complete datasets.

Numerical interview items (Q1‐Q4‐Q6‐Q8‐Q11‐Q15‐Q17‐Q19‐Q21) were averaged to obtain a composite socio‐occupational functioning score (Q_mean), used as a continuous descriptive index. For regression analyses, a reduced score including only items available for all participants (excluding Q4 and Q11) was used, ensuring consistency of the outcome measure across subjects.

Internal consistency was assessed using Cronbach's alpha.

Associations between Q_mean and demographic or clinical variables were first screened using univariate linear regression analyses, considering age, sex, disease duration, education level, genetic status, and the percentage pre‐post change (Δ = [(post‐pre)/pre] × 100) of MDS‐UPDRS III, axial subscores, LEDD, MMSE, BDI, STAI‐X1, and AS. No formal correction for multiple comparisons was applied in the primary screening analysis, as these analyses were intended for exploratory variable selection; false discovery rate (FDR) correction was added as a sensitivity analysis. Variables with p‐value<0.05 in univariate screening were entered into a multivariate linear regression model to identify independent predictors of Q_mean with the number of predictors limited to a maximum of three given the sample size. Model stability was further assessed using leave‐one‐out cross‐validation (LOO‐CV), and cross‐validated R^2^ was calculated.

Analyses were performed using SPSS 29, with two‐tailed significance at *P* = 0.05.

## Results

Of 40 patients screened, six were excluded (n = 3 incomplete data, n = 2 in follow‐up at another center, and one for postoperative dementia). Thirty‐four patients (27 males; age 59.8 ± 7.7 years; disease duration 11.7 ± 2.5 years) completed the socio‐occupational interview at 13.9 ± 3.5 months post‐surgery. Pathogenic variants in major PD‐related genes were identified in five patients (14.7%), all carriers of a heterozygous *GBA* variant.

STN‐DBS was associated with a significant reduction in OFF‐MED MDS‐UPDRS III scores (−29.8%, *P* = 0.001), LEDD (−50%, *P* = 0.001) and MDS‐UPDRS IV (−49.2%, *P* = 0.001). PDQ‐39 also improved in domains of mobility, stigma, ADL and body discomfort (all *P* = 0.05). MMSE, BDI, and STAI‐X2 scores remained stable, while STAI‐X1 showed a non‐significant postoperative increase (*P* = 0.065), whereas apathy significantly increased (*P* = 0.017) (Supplementary Table S1).

### Socio‐Occupational Functioning

Participants reported overall favorable functioning (Table 1, Supplementary Fig. S1). The Q_mean score was 7.66 ± 0.86 (range 6–9.57). Higher ratings were observed for perceived DBS efficacy, general well‐being, and family relationships, while greater variability was noted for hobbies and work‐related satisfaction.

**TABLE 1:** Patient answers to the socio‐occupational interview

Item	Response type	N (valid)	Score	N Yes (%)
Satisfaction with STN‐DBS surgery	0–10 scale	34	8.4 ± 1.2 (6–10)	‐
2 Working before STN‐DBS	Yes/No	34	‐	20 (18 M/2 W) (58.8%)
3 Discontinued work after STN‐DBS	Yes/No	20	‐	3 (3 M) (15%)
3A. Changed job	0–10 scale	17	‐	10 (9 M/1 W) (58.8%)
4 Work satisfaction	0–10 scale	17	6.9 ± 1.8 (4–10)	‐
5 Able to pursue hobbies/interests	Yes/No	34	‐	25 (18 M/7 W) (73.5%)
6 Satisfaction with hobbies/interests	0–10 scale	34	7 ± 1.8 (1–10)	‐
7 Able to perform domestic activities	Yes/No	34	‐	28 (22 M/6 W) (82.4%)
8 Satisfaction with domestic activities	0–10 scale	34	7.4 ± 1.5 (5–10)	‐
9 Able to achieve short/long‐term goals more easily	Yes/No	34	‐	24 (19 M/5 W) (70.6%)
10 Need of assistance in daily activities	Yes/No	34	‐	9 (6 M/3 W) (26.5%)
11 Satisfaction with assistance/support received	0–10 scale	9	8.6 ± 1 (7–10)	‐
12 Current reference figures (family/friends)	Open‐ended	34	‐	‐
13 Change in reference figures after STN‐DBS	Yes/No	34	‐	3 (3 M) (8.8%)
14 Loss of an important relationship	Yes/No	34	‐	3 (3 M) (8.8%)
15 Satisfaction with family relationships	0–10 scale	34	7.9 ± 1.3 (5–10)	‐
16 Able to maintain/develop social friendships	Yes/No	34	‐	30 (23 M/7 W) (88.2%)
17 Satisfaction with social/friendship relations	0–10 scale	34	7.6 ± 1.4 (5–10)	‐
18 Change in daily routine management	Yes/No	34	‐	27 (21 M/7 W) (79.4%)
19 Satisfaction with daily routine management	0–10 scale	34	7.6 ± 1.1 (6–10)	‐
20 Improvement in general well‐being	Yes/No	34	‐	26 (20 M/6 W) (76.5%)
21 Perceived improvement in well‐being	0–10 scale	34	7.7 ± 1.3 (5–10)	‐

*Note*: Results are presented as Mean ± Standard deviation (Range), or Absolute values (Percentage), as appropriate.

Abbreviation: M, Men; STN‐DBS, subthalamic nucleus deep brain stimulation; W, Women.

Among 20 previously employed patients, three stopped working (two due to retirement) and 10 modified job duties. Cronbach's alpha for the seven numerical items available for all participants (excluding Q4 and Q11, not answered by all participants) was 0.769, supporting internal coherence as a summary index of related socio‐occupational domains, without implying construct validation. This reduced Q_mean (7.66 ± 0.91) was used for regression analyses.

### Exploratory Evaluation of Factors Associated with Socio‐Occupational Functioning

**Figure 1:**
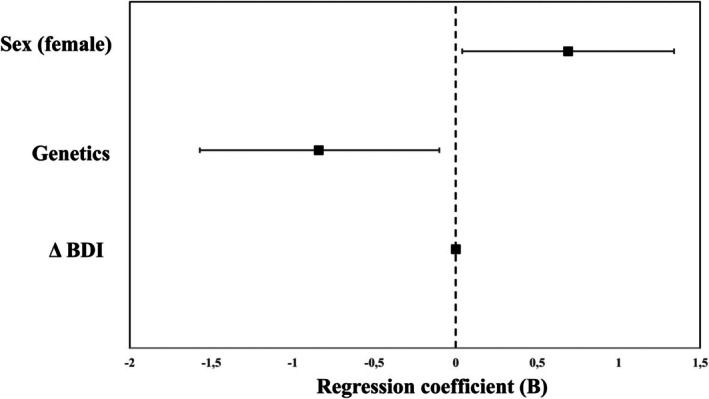
Associations Between Clinical Variables and Socio‐Occupational Functioning (Q_mean). Displayed values derive from the multivariate regression analysis. Detailed parameter estimates and specific Odds Ratio are reported in Supplementary Table S2. Unstandardized regression coefficients (B) with 95% confidence intervals from the multivariate linear regression model are shown in the plot. Detailed parameters estimates and specific coefficients are reported in supplementary Table S2. Legend: Δ = percentage pre‐post surgery change [(post‐pre)/pre] × 100; BDI, Beck Depression Inventory; MDS‐UPDRS, Movement Disorder Society Unified Parkinson's Disease Rating Scale.

In univariate screening analysis, Q_mean was associated with sex (*P* = 0.020), genetic status (*P* = 0.006), ΔBDI (*P* = 0.013), ΔMDS‐UPDRS IV (*P* = 0.036), and ΔAxial_ON (*P* = 0.022). After FDR correction, none of these associations remained statistically significant. In the exploratory multivariate model including sex, genetics, and ΔBDI, the overall fit was significant (*F*_(3,30)_ = 8.52, *P* = 0.001; adjusted R^2^ = 0.406; LOO‐CV *R* ^2^ = 0.29) (Supplementary Table S2). Female sex (β = 0.449, *P* = 0.003) and genetic status (β = −0.446, *P* = 0.007) showed independent associations with socio‐occupational functioning, with higher Q_mean scores in women and lower scores in *GBA* variant carriers (Fig. 1), whereas mood change showed a positive but nonsignificant trend.

## Discussion

In this single‐center cohort of PD patients assessed 9–18 months after STN‐DBS, socio‐occupational functioning appeared overall satisfactory. Most participants reported good social engagement and daily‐life interactions, suggesting that some aspects of everyday functioning after STN‐DBS may not be fully captured by standard motor, non‐motor or QoL scales.^2^, ^3^, ^4^, ^5^

The semi‐structured interview used in this study enabled a focused assessment of social and occupational dimensions often underrepresented in routine DBS follow‐up. At the same time, it captures patients’ perceived level of functioning at a defined postoperative time point rather than longitudinal changes. The lack of significant changes in standardized psychosocial scales such as the BDI, STAI, or selected PDQ‐39 subdomains does not necessarily contradict these findings, as these instruments are not designed to specifically capture role functioning, occupational satisfaction, or perceived social participation. Moreover, apathy significantly increased, as previously reported in other STN‐DBS cohorts,^9^ and, although not independently associated with Q_mean, its worsening may still influence daily motivation and engagement, potentially moderating real‐life adaptation after surgery.

The composite Q_mean score should be interpreted as a pragmatic summary index of patient‐perceived functioning rather than a validated scale. Its purpose was to facilitate quantitative exploration of socio‐occupational dimensions in the absence of established instruments, without implying construct validation or defining thresholds of impairment or recovery. Internal consistency was acceptable, supporting its use as a descriptive index of related domains.

In the exploratory multivariate analyses, sex and genetic status (presence of a pathogenic *GBA* variant) showed independent associations with Q_mean. These findings should be regarded as hypothesis‐generating rather than robust or generalizable conclusions, given the limited number of women (n = 7) and *GBA* carriers (n = 5), and the lower cross‐validated R^2^ compared with the apparent model fit. Nonetheless, the results provide possible directions for future research. The higher perceived functioning reported by women may reflect differences in subjective social functioning rather than objective occupational outcomes, as opportunities for occupational reintegration were limited in this subgroup. Previous studies have suggested sex‐related differences in DBS outcomes, including psychosocial domains,^10^, ^11^ but the present data are insufficient to draw firm conclusions.

Conversely, the negative association between GBA status and socio‐occupational functioning is consistent with evidence that genetic background may influence DBS outcomes. While large multicenter studies suggest that DBS does not exacerbate cognitive decline in GBA‐associated PD,^12^, ^13^ our findings may contribute to discussions on potential sources of variability in patient‐reported outcomes after DBS.

More broadly, the lack of an independent association between socio‐occupational functioning and motor outcomes suggests that post‐DBS adjustment may depend on multidimensional factors beyond motor improvement alone. Non‐motor features, contextual factors, and subjective perceptions may all contribute to social reintegration after surgery, underscoring the value of multidisciplinary follow‐up approaches.

In addition, sociocultural and healthcare system factors may influence real‐life functional outcomes. Our cohort was evaluated within the Italian healthcare context, characterized by universal health coverage and disability support systems, which may affect occupational reintegration and social participation after surgery. Therefore, the generalizability of these findings to healthcare systems with different support structures should be interpreted with caution.

Several limitations must be acknowledged. The small sample size and single‐center limit generalizability. Socio‐occupational functioning was assessed using a non‐validated semi‐structured interview and only postoperatively; therefore, we cannot determine whether the observed levels of functioning reflect changes after surgery or pre‐existing functioning. Third, the follow‐up window captures early postoperative adaptation but not long‐term outcomes. Finally, the limited number of women and *GBA* carriers substantially restricts interpretation of subgroup findings.

Despite these limitations, the study contributes to the growing literature addressing social and occupational dimensions of DBS outcomes. Future multicenter investigations, including larger cohorts evaluated at different stages of the DBS trajectory and with standardized psychosocial instruments, will be important to better delineate predictors of real‐life functioning after STN‐DBS.

## Author Roles

(1) Research project: A. Conception, B. Organization, C. Execution; (2) Statistical Analysis: A. Design, B. Execution, C. Review and Critique; (3) Manuscript Preparation: A. Writing of the first draft, B. Review and Critique.

G.I.: 1B, 1C, 2A, 2B, 3A.

E.M.: 1A, 1B, 1C, 2C, 3B.

C.L.: 1C, 2C, 3B.

M.G.: 1C, 2C, 3B.

A.R.: 1C, 2C, 3B.

M.G.R.: 1C, 2C, 3B.

L.L.: 1A, 1B, 1C, 2C, 3B.

M.Z.: 1A, 1B, 1C, 2A, 2C, 3B.

## Disclosures

**Ethical Compliance Statement:** The aim of the study was explained to the participants, and written informed consent was obtained before the study was initiated Approval was obtained for the study from the AOU Citta della Salute e della Scienza Torino review board. The study was conducted according to the ethical standards of the 1964 Declaration of Helsinki. We confirm that we have read the Journal's position on issues involved in ethical publication and affirm that this work is consistent with those guidelines.

**Funding Sources and Conflict of Interest:** This study was partly performed under the Department of Excellence grant of the Italian Ministry of Education, University and Research to the “Rita Levi Montalcini” Department of Neuroscience, University of Torino, Italy. The authors declare that they have no known competing financial interests or personal relationships that could have appeared to influence the work reported in this paper.

**Financial Disclosures for the Previous 12 Months:** GI received speaker honoraria from Bial, Abbvie. LL received speaker honoraria from Bial, Abbvie, Zambon, Ralpharma. AR, MGR and MZ received speaker honoraria from Zambon, Bial, AbbVie. No additional disclosures to report.

## Financial Disclosures and Conflicts of Interest

Author disclosures are available in the Supporting Information.

## Supporting information


**File S1.** The semi‐structured interview. This document is the English translation of the semi‐structured interview administered (in Italian language) to the patients to explore perceived changes in occupational, domestic, and social functioning after STN‐DBS.
**TABLE S1.** Demographic and clinical values of screened and enrolled patients at baseline (before surgery) and at 9–18 months after surgery.
**Figure S1.** Multidimensional socio‐occupational functioning assessed through the semi‐structured interview. Values in the scatter plot represent mean socio‐occupational functioning scores; detailed item‐level data are reported in Table 1 (0–10 scores for the nine items with numerical rating: Q1, Q4, Q6, Q8, Q11, Q15, Q17, Q19, Q21).
**TABLE S2.** Multivariate analysis of the association between patients' characteristics and mean value of interview.

**Data S1.** ICJME_paper socio‐occupational functioning DBS.

## Data Availability

The data that support the findings of this study are available on request from the corresponding author. The data are not publicly available due to privacy or ethical restrictions.
