# Supplementary material for: Socio‐Occupational Functioning after Subthalamic Deep Brain Stimulation in Parkinson's Disease
Source: Mov Disord Clin Pract. 2026 May 1;13(10):2546–51. doi: 10.1002/mdc3.70661 (PMC13339451; doi:10.1002/mdc3.70661)
Supplement: Supplementary file 2 [file MDC3-13-2546-s002.docx]

**SUPPLEMENTAL FILES**

**Supplemental File 1. The semi-structured interview.**

*We are conducting a research study to explore possible changes in family dynamics and socio-occupational functioning following deep brain stimulation (DBS) neurosurgery for the treatment of Parkinson’s disease.*

*The study involves answering a number of questions through a semi-structured interview.
We kindly ask you to provide your consent and to answer spontaneously and sincerely. Please note that your responses will in no way interfere either with the treatments you are receiving or with your relationship with your healthcare providers.*

Date of administration	………..………………………………………………………………..

**SOCIODEMOGRAPHIC DATA**

1) Patient initials	...…….……….…………………………………………………………
2) Gender

□	Male

□	Female

3) Age	…………………………………………………………………………
4) Date of birth	…………………………………………………………………………
5) Place of birth	…………………………………………………………………………
6) Place of residence	………………………………………………………………………….
7) Education (years of schooling) ……………………………………………………………………
8) Employment status	…………………………………………………………………………

□	Employed

□	Unemployed

□	Retired

9) Marital status

□	Single	□ Divorced

□	Married	□ Cohabiting

□	Widowed

**INTERVIEW**

1) On a scale from 1 to 10, how satisfied are you with the DBS surgery?

……………………………………………………………………………………………………

2) Before undergoing DBS surgery, were you employed?

Yes	□

No	□

3) After the DBS surgery, did you stop working?

Yes	□

No	□

If no, have your work activities and/or duties changed?

Yes	□

No	□

4) On a scale from 1 to 10, how satisfied are you with the performance of your work activities?

……………………………………………………………………………………………………

5) After the DBS surgery, have you been able to pursue your interests/hobbies?

Yes	□

No	□

6) On a scale from 1 to 10, how satisfied are you with your ability to pursue your interests/hobbies?

……………………………………………………………………………………………………

7) After the DBS surgery, have you been able to carry out daily household activities? (e.g. managing the home, shopping, etc.)

Yes	□

No	□

8) On a scale from 1 to 10, how satisfied are you with the management of your daily household activities?

……………………………………………………………………………………………………

9) After the DBS surgery, do you find it easier to achieve your short- and long-term goals?

Yes	□

No	□

10) After the DBS surgery, do you need assistance or support with activities of daily living?

Yes	□

No	□

11) On a scale from 1 to 10, how satisfied are you with the assistance or support you receive?

…………………………………………………………………………………………………

12) Who are your current reference persons (within your family and friendship circle)?

……………………………………………………………………………………………………

13) After the DBS surgery, have your reference persons changed?

Yes	□

No	□

14) After the DBS surgery, have you experienced the loss of an important relationship?

Yes	□

No	□

If yes, please specify (e.g., bereavement, separation, divorce)……………………………………

15) On a scale from 1 to 10, how satisfied are you with your relationships with family members?

……………………………………………………………………………………………………

16) After the DBS surgery, have you been able to maintain/develop social/friendship relationships?

Yes	□

No	□

17) On a scale from 1 to 10, how satisfied are you with your social/friendship relationships?

……………………………………………………………………………………………………

18) After the DBS surgery, do you think the management of your daily routine has changed?

Yes	□

No	□

19) On a scale from 1 to 10, how satisfied are you with the management of your daily routine?

……………………………………………………………………………………………………

20) Do you believe that your general well-being has improved after DBS surgery?

Yes	□

No	□

21) On a scale from 1 to 10, how much has your general well-being improved following DBS?

……………………………………………………………………………………………………

**Supplementary Figure 1. Multidimensional socio-occupational functioning assessed through the semi-structured interview.**

Values in the scatter plot represent mean socio-occupational functioning scores; detailed item-level data are reported in Table 1 (0-10 scores for the nine items with numerical rating: Q1, Q4, Q6, Q8, Q11, Q15, Q17, Q19, Q21).

**Legend:** STN-DBS: Subthalamic Nucleus Deep Brain Stimulation

**Supplementary Table 1. Demographic and clinical values of screened and enrolled patients at baseline (before surgery) and at 9-18 months after surgery**

|  | **Screened Patients (n=40)** | **Pre-DBS (n=34)** | **Post-DBS (n=34)** | **P value** |
| --- | --- | --- | --- | --- |
| **Age (years)** | 59.6 ± 7.6 | 59.8 ± 7.7 | / | / |
| **Sex (M/W)** | (31/9) | (27/7) | / | / |
| **Instruction (Years)** | 13.2 ± 4.1 | 13.3 ± 4.2 | / | / |
| **Disease Duration (years)** | 11.9 ± 2.6 | 11.7 ± 2.5 | / | / |
| **STN-DBS duration (months)** | / | / | 13.9 ± 3.5 | / |
| **LEDD total** | 1217.3 ± 353.3 | 1205.5 ± 319 | 663.7 ± 313.7 | ***< 0.001*** |
| **LEDD D-agonist** | 182.6 ± 156.7 | 185.1 ± 152.1 | 87.1 ± 90.8 | ***< 0.001*** |
| **MDS-UPDRS I** | 13.5 ± 5.0 | 13.1 ± 5.6 | 12.1 ± 5.5 | 0.437 |
| **MDS-UPDRS II** | 17 ± 6.6 | 15.8 ± 7.4 | 13.6 ± 6.8 | 0.099 |
| **MDS-UPDRS III OFF MED** | 47.3 ± 13.3 | 46.1 ± 13.9 | 31.7 ± 13.7 | ***< 0.001*** |
| **MDS-UPDRS III ON MED** | 18.0 ± 9.8 | 17.5 ± 10.2 | 14.9 ± 9 | 0.064 |
| **HY OFF MED** | 2.3 ± 0.7 | 2.3 ± 0.6 | 2.2 ± 0.6 | 0.617 |
| **HY ON MED** | 1.9 ± 0.6 | 1.8 ± 0.5 | 1.9 ± 0.6 | 0.317 |
| **AXIAL OFF MED** | 8.4 ± 4.2 | 8.4 ± 4.2 | 5.9 ± 4.7 | ***< 0.001*** |
| **AXIAL ON MED** | 3.2 ± 2.1 | 3.4 ± 2.3 | 3.2 ± 3.1 | 0.599 |
| **MDS-UPDRS IV** | 10.2 ± 3.4 | 10.2 ± 3.5 | 4.6 ± 3.3 | ***< 0.001*** |
| **MMSE** | 28.8 ± 1.2 | 28.8 ± 1.2 | 29.2 ± 1.1 | 0.102 |
| **BDI** | 9.7 ± 5.3 | 10 ± 5.2 | 9.4 ± 5.5 | 0.343 |
| **STAIX1** | 39.8 ± 7.6 | 39.6 ± 7.4 | 40.2 ± 7.4 | 0.065 |
| **STAIX2** | 40.6 ± 7.8 | 40.5 ± 7.5 | 40.4 ± 7.6 | 0.381 |
| **AS** | 10.6 ± 5.2 | 11.1 ± 4.2 | 12.9 ± 5.8 | **0.017** |
| **PDQ-39 Tot (%)** | 24.8 ± 12.4 | 24.3 ± 9.1 | 20.5 ± 8.6 | ***0.028*** |
| **PDQ-39 mobility (%)** | 24.8 ± 15.5 | 26.2 ± 15.6 | 22.3 ± 17.5 | ***0.006*** |
| **PDQ-39 ADL (%)** | 28.4 ± 19.4 | 28.9 ± 19.2 | 19.7 ± 14.7 | ***< 0.001*** |
| **PDQ-39 emotions (%)** | 20.5 ± 11.3 | 21.8 ± 11 | 21.9 ± 14.8 | 0.940 |
| **PDQ-39 stigma (%)** | 30.8 ± 16.9 | 33.2 ± 16 | 26.7 ± 12.8 | ***0.017*** |
| **PDQ-39 social support (%)** | 13.5 ± 14.1 | 14 ± 13.4 | 11.5 ± 9.8 | 0.732 |
| **PDQ-39 cognition (%)** | 17.7 ± 11.7 | 18.8 ± 10.8 | 16.5 ± 9 | 0.256 |
| **PDQ-39 communication (%)** | 14.5 ± 11.7 | 16.6 ± 12 | 19.4 ± 16.8 | 0.239 |
| **PDQ-39 body discomfort (%)** | 39.2 ± 22.8 | 37.3 ± 20.8 | 27 ± 22 | ***0.012*** |

Results are presented as mean ± standard deviation or absolute values, as appropriate. P values are presented for Wilcoxon signed-rank test of 34 patients evaluated before and after surgery.

**Legend:** AS: Marin Apathy Scale; Axial: sum of MDS-UPDRS items 3.1, 3.3, 3.9, and 3.10-3.13; BDI: Beck Depression Inventory; HY: Hoehn and Yahr; LEDD: Levodopa equivalent daily dose; M: Men; MMSE: Mini Mental State Examination; MDS-UPDRS: Movement Disorder Society Unified Parkinson's Disease Rating Scale; PDQ-39: Parkinson's Disease Questionnaire; STAI: State-Trait Anxiety Inventory: STN-DBS: Subthalamic Nucleus Deep Brain Stimulation; W: Women.

**Supplementary table 2. Multivariate analysis of the association between patients’ characteristics and mean value of interview**

| **Predictor** | **B (SE)** | **β** | **t** | **p** | **95% CI for B** |
| --- | --- | --- | --- | --- | --- |
| **Constant** | 6.71 (0.36) | — | 18.58 | <0.001 | 5.97 – 7.45 |
| **Sex (Female)** | 0.94 (0.29) | 0.45 | 3.27 | 0.003 | 0.35 – 1.53 |
| **Genetic status (GBA+)** | –0.99 (0.35) | –0.45 | –2.88 | 0.007 | -1.69 – -0.29 |
| **Δ BDI** | 0.001 (0.001) | 0.18 | 1.16 | 0.256 | -0.001 – 0.003 |

**Model summary:** R = 0.678, R² = 0.460, adjusted R² = 0.406, F(3,30) = 8.52, p < 0.001, Durbin–Watson = 1.99.

Legend: Δ = percentage pre-post surgery change [(post-pre)/pre] x 100; Axial: sum of MDS-UPDRS items 3.1, 3.3, 3.9, and 3.10-3.13; BDI: Beck Depression Inventory; CI: Confidence Interval; MDS-UPDRS: Movement Disorder Society Unified Parkinson's Disease Rating Scale; SE: Standard Error.
